# Accurate and Fast Convergent Initial-Value Belief Propagation for Stereo Matching

**DOI:** 10.1371/journal.pone.0137530

**Published:** 2015-09-08

**Authors:** Xiaofeng Wang, Yiguang Liu

**Affiliations:** 1 Vision and Image Processing Laboratory, College of Computer Science, Sichuan University, Chengdu, P.R. China; 2 College of Mathematical and Physical Sciences, Chongqing University of Science and Technology, Chongqing, P.R. China; Institute of Automation, Chinese Academy of Sciences, CHINA

## Abstract

The belief propagation (BP) algorithm has some limitations, including ambiguous edges and textureless regions, and slow convergence speed. To address these problems, we present a novel algorithm that intrinsically improves both the accuracy and the convergence speed of BP. First, traditional BP generally consumes time due to numerous iterations. To reduce the number of iterations, inspired by the crucial importance of the initial value in nonlinear problems, a novel initial-value belief propagation (IVBP) algorithm is presented, which can greatly improve both convergence speed and accuracy. Second, .the majority of the existing research on BP concentrates on the smoothness term or other energy terms, neglecting the significance of the data term. In this study, a self-adapting dissimilarity data term (SDDT) is presented to improve the accuracy of the data term, which incorporates an additional gradient-based measure into the traditional data term, with the weight determined by the robust measure-based control function. Finally, this study explores the effective combination of local methods and global methods. The experimental results have demonstrated that our method performs well compared with the state-of-the-art BP and simultaneously holds better edge-preserving smoothing effects with fast convergence speed in the Middlebury and new 2014 Middlebury datasets.

## Introduction

Stereo matching is one of the most extensively researched topics in computer vision and aims to infer a dense disparity or depth map by finding the correct correspondence between a pair of images (inference image and target image) captured from different viewpoints or at different times. In recent decades, many high-quality studies have been conducted [1, 2, 3].

Generally, stereo matching problems can be classified into global methods and local methods [4, 5]. For global methods, stereo matching is commonly formulated as energy function minimization frameworks. The belief propagation (BP) [6, 7] algorithm is one of the most popular global methods [8]. Numerous methods have been presented to improve BP, including loopy belief propagation (LBP) [7], hierarchical belief propagation (HBP) [9], context guided BP (CBP) [10], and fast-converging belief propagation [1]. However, these methods are primarily designed to decrease time consumption without intrinsically improving the accuracy of BP. Although some fast BP algorithms rise, the BP algorithm is still much slower than local methods, preventing it from meeting real-time requirements. In addition, BP also exist some problems, such as the Markov random field (MRF) shrinking bias, over-smoothness phenomenon, and ambiguous edges of the disparity map [11]. Relatively, local methods often compute disparity values within a finite region. Traditional local methods are typically faster than global methods with lower accuracy. Recently, some excellent local algorithms have produced and performances are similar to those of state-of-the-art global algorithms, such as the bilateral filter (BF) [12] and guided filter (GF) [13, 14]. Therefore, combining the advantages of local methods will provide a more beneficial reference to improve the accuracy and efficiency of BP.

The main contribution of this paper lies in presenting an initial-value belief propagation (IVBP) with high accuracy and fast convergence, inspired by the importance of the initial value for complex nonlinear problems. A more accurate initial value is helpful in acquiring a more accurate solution of the nonlinear problem, along with the much faster convergence velocity. Similarly to the nonlinear problem, BP is also a cyclic iterative method. An accurate initial value can cause fast convergence of BP while acquiring more accurate disparity values. Local methods with both good accuracy and high convergence speed are especially important for the performance of BP. Presently, GF is one of the best local approaches in terms of accuracy and speed. The disparity map of GF can be set as the initial value of BP, providing different disparities with different probabilities based on the initial value of the local methods instead of traditional equal probabilities. Based on this idea, IVBP is proposed.

Another contribution of the paper is the successful refining of the data term of the BP algorithm. In the past decade, many improved BP algorithms have been reported [1,7,9]. However, these methods concentrate on the smoothness term or other energy terms rather than the data term, and they neglect the importance of the data term for accuracy and speed to a certain degree. In fact, there is a relationship and discrimination between the data and smoothness terms. The reference accuracy of the smoothness term and the other energy terms greatly depends on the fidelity of the data term. Improvement of the data term can simultaneously be helpful for both the accuracy and convergence of BP. Therefore, in this paper, a self-adapting dissimilarity data term (SDDT) is proposed instead of the traditional data term, combining the intensity-based measure and gradient-based measure with contextual inference using a robust measure-based control function. This method also effectively improves the performance in textureless regions and near discontinuities regions.

This study also includes an exploration of the combination of local methods and global methods. We find that local methods and global methods have different advantages and disadvantages; e.g., local methods are simple and fast, whereas global methods are relatively complex and slow. Local methods primarily depend on disparity values of the local window and perform poorly in textureless and occluded regions, whereas global methods perform better in these regions due to information inference. Therefore, their combination should result in more competitive performance, which is considered as important as establishment of a new method. Further, we also expect that this study will attract more attention to the exploration of fusions of local and global methods.

The rest of this paper is organized as follows: in Section 2, we provide a brief overview of BP and then propose IVBP. Section 3 discusses experiments to evaluate the proposed methods, demonstrating the effectiveness of our optimization framework on the Middlebury and new 2014 Middlebury datasets. Finally, Section 4 provides some conclusions.

## Our Algorithm

In this paper, to improve accuracy and speed, we present an accurate and fast-converging IVBP.

**Fig 1:**
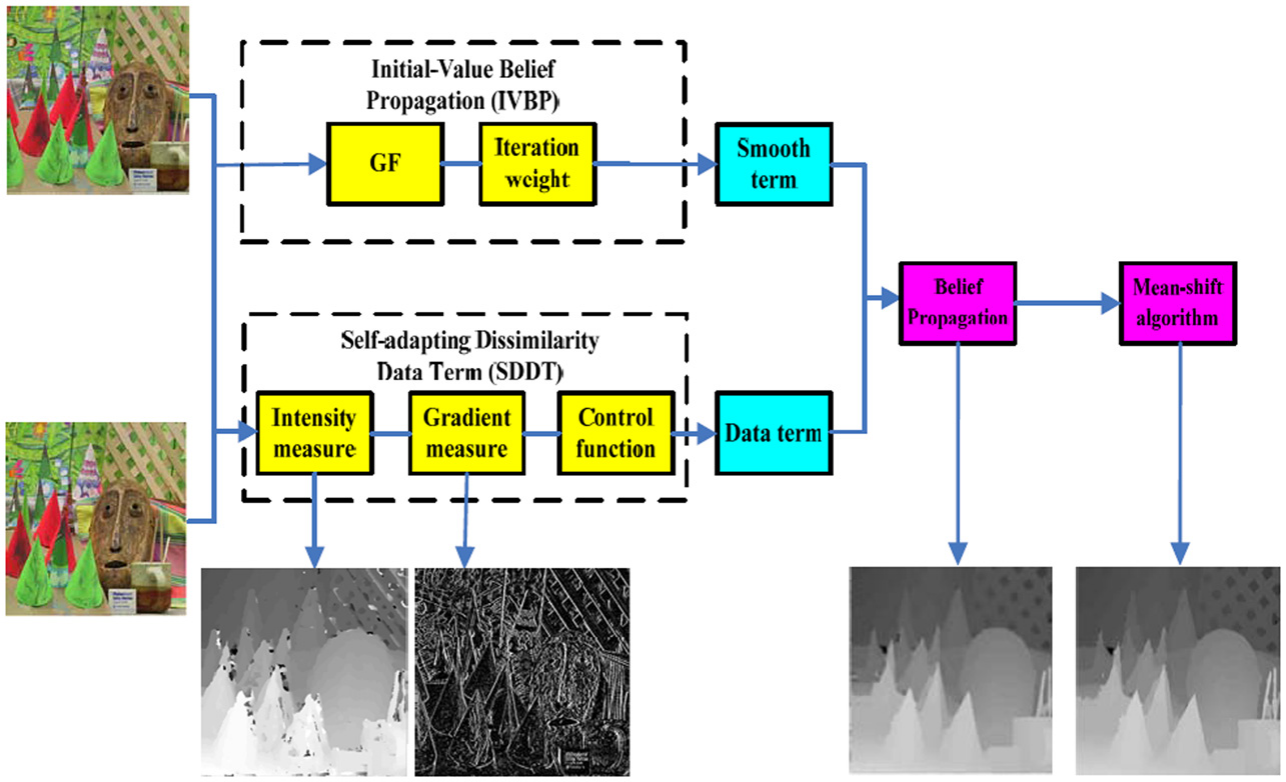
Block diagram of our algorithm. See the text for more details.

First, we briefly provide an overview of our methods. Fig 1 depicts this overview. Second, we propose SDDT, which combines intensity-based measure and gradient-based measure using a robust measure-based control function in Section 2.2. Last, we present IVBP in Section 2.3.

### Belief Propagation

Stereo correspondence can be formulated as the estimation of disparity values for every node in an MPF using BP. This section briefly reviews the BP adopted here. BP is an inference algorithm used to find the most likely disparity value of each node through a global energy-minimization framework [8, 9] and is defined as follows:

Let *I* be a set of pixels and *D* be a finite set of label maps with respect to *I*. Our aim is searching for the mapping *ℓ*:*I*→*D* that assigns each pixel a reliable disparity value. (*p*,*q*) represents a pair of neighboring nodes to a neighborhood *N*(·). A label map *f* ∈ *ℓ* assigns (*f*_*p*_,*f*_*q*_) ∈ *D* to each pixel (*p*,*q*) ∈ *I*. The compatibility function Φ(*p*,*f*_*p*_) denotes how compatible a disparity value *f*_*p*_ is with the intensity value *p* observed in the images, and the evidence function Ψ(*f*_*p*_,*f*_*q*_) denotes differences of two disparity pairs (*f*_*p*_,*f*_*q*_).

Considering the compatibility of neighboring disparity variables (*f*_*p*_,*f*_*q*_) and intensity values (*p*,*q*) ∈ *D* between the image pairs, the joint posterior probability of MRF can be defined as [8]:

$$P(D|I)\propto \underset{(p,q)\in N}{\prod }\Psi (f_{p},f_{q})\underset{p\in P}{\prod }\Phi (p,f_{P}),\;\forall (p,q)\in I$$ (1)

Through the-log of Eq (1), the above equation *P*(·) based on maximizing the probability of MRF is equivalent to minimizing the following energy function *E*(·):

$$E(P(D|I))\propto \underset{(p,q)\in N}{\sum }-\text{log}\Psi (f_{p},f_{q})+\underset{p\in P}{\sum }-\text{log}\Phi (p,f_{p}).$$ (2)

We simplify *E*(*P*(*D*|*I*)) using *E*(*D*|*I*), and thus Eq (2) can be expressed as:

$$E(D|I)\propto V(f_{p},f_{q})+D(p,f_{p})$$ (3)

where the functions *V*(·) and *D*(·) represent the data and smoothness terms, respectively.

### Self-Adapting Dissimilarity Data Term

As described previously, BP mainly consists of two terms: the data term *D*(·) and smoothness term *V*(·). Based on a lot of experiment, it is found that the data term *D*(·) is important for the accuracy of BP. Therefore, to improve the accuracy of the data term, we present an SDDT.

#### Self-adapting Dissimilarity Data Term

Often, the data term depends on pixel-based intensity measures, such as absolute intensity differences (AD) and squared intensity differences (SD),etc. A major limitation of the above-mentioned methods is that they utilize only raw intensity [15]. That is because the raw intensity measure does not consider contextual constraints and depending only on raw intensity is challenging in locally ambiguous regions, such as discontinuous and textureless regions. Relatively, gradient-based measures can provide contextual constraints. Therefore, combining intensity-based measures and gradient-based measures should acquire the better performance compared with individual intensity-based measures, similarly to the self-adapting dissimilarity measure in [16]. However, Andreas Klaus only combined the self-adapting dissimilarity measure with color segmentation on the reference image without essentially combining global methods. In contrast with previous work [16], we incorporated the self-adapting dissimilarity measure into the data term of BP for global methods and propose SDDT.

Here, the data term *D*(·) adopted in this study is briefly reviewed. Suppose that *I*_*L*_(·) and *I*_*R*_(·) are a reference image and target image, respectively. Let *D*_*AD*_(*x*,*y*,*d*) represent the AD measure at position (*x*,*y*) with disparity value *d*, where *N*(*x*,*y*) represents a neighborhood at position (*x*,*y*), *N*_*X*_(*x*,*y*) represents a neighborhood without a right-most column, *N*_*Y*_(*x*,*y*) represents a surrounding window without the lowest column, ∇_*X*_ represents the central difference to the right, and ∇_*Y*_ is the central difference to the bottom. Then, the AD measure *D*_*AD*_(·) is computed as follows:

$$D_{AD}(x,y,d)=\underset{\left(x_{k},y_{k})\in N(x,y\right)}{\sum }\left|I_{L}(x_{k},y_{k})-I_{R}(x_{k}+d,y_{k})\right|.$$ (4)

Next, SDDT is proposed to improve both accuracy and robustness. Let *D*_*GRAD*_(*x*,*y*,*d*) represent the gradient-based measure at position (*x*,*y*) with disparity value *d*. Then, the gradient-based measure *D*_*GRAD*_(·) is defined as follows:

$$D_{GRAD}(x,y,d)=\underset{\left(x_{k},y_{k})\in N_{X}(x,y\right)}{\sum }\left|\nabla_{X}I_{L}(x_{k},y_{k})-\nabla_{X}I_{R}(x_{k}+d,y_{k})\right|+\underset{\left(x_{k},y_{k})\in N_{Y}(x,y\right)}{\sum }\left|\nabla_{Y}I_{L}(x_{k},y_{k})-\nabla_{Y}I_{R}(x_{k}+d,y_{k})\right|.$$ (5)

Further, to balance two previous measures, the optimal weighting ϖ between intensity-based measure *D*_*AD*_(·) and gradient-based measure *D*_*GRAD*_(·) is defined. The improved data term *D*′(·) is defined as follows:

$$D^{\prime }(x,y,d)=(1-\varpi )D_{AD}(x,y,d)+\varpi D_{GRAD}(x,y,d).$$ (6)

Note that the improved data term contains the traditional data term as a special case, when the optimal weighting ϖ=0.

### Robust Control Function

For data term *D*′(·), experiments demonstrate that there is inevitably a little or even severe bias between *D*_*AD*_(·) and *D*_*GRAD*_(·). To further avoid the bias of the two measures, the simple but effective robust control function *ρ*(·) is proposed.

Through *ρ*(*c*,*λ*), different cost measures can be mapped to the range [0, 1]. Therefore, it can control the influence of the outliers by adjusting the parameter *λ*. The SDDT is further described in Eq (7):

$$D^{\prime \prime }(x,y,d)=(1-\varpi^{\prime })\rho (D_{AD}(x,y,d),\lambda_{AD})+\varpi^{\prime }\rho (D_{GRAD}(x,y,d),\lambda_{GRAD}),$$ (7)

Where *ρ*(*D*(·),*λ*) = 1 –exp(−*D*(·)/*λ*).

Here, $\varpi^{\prime }$ is determined as the optimal weighting between *ρ*(*D*_*AD*_(·)) and *ρ*(*D*_*GRAD*_(·)). See Section 3.1 for the discussion of the parameter setting $\varpi^{\prime }$ and its sensitivity.

**Fig 2:**
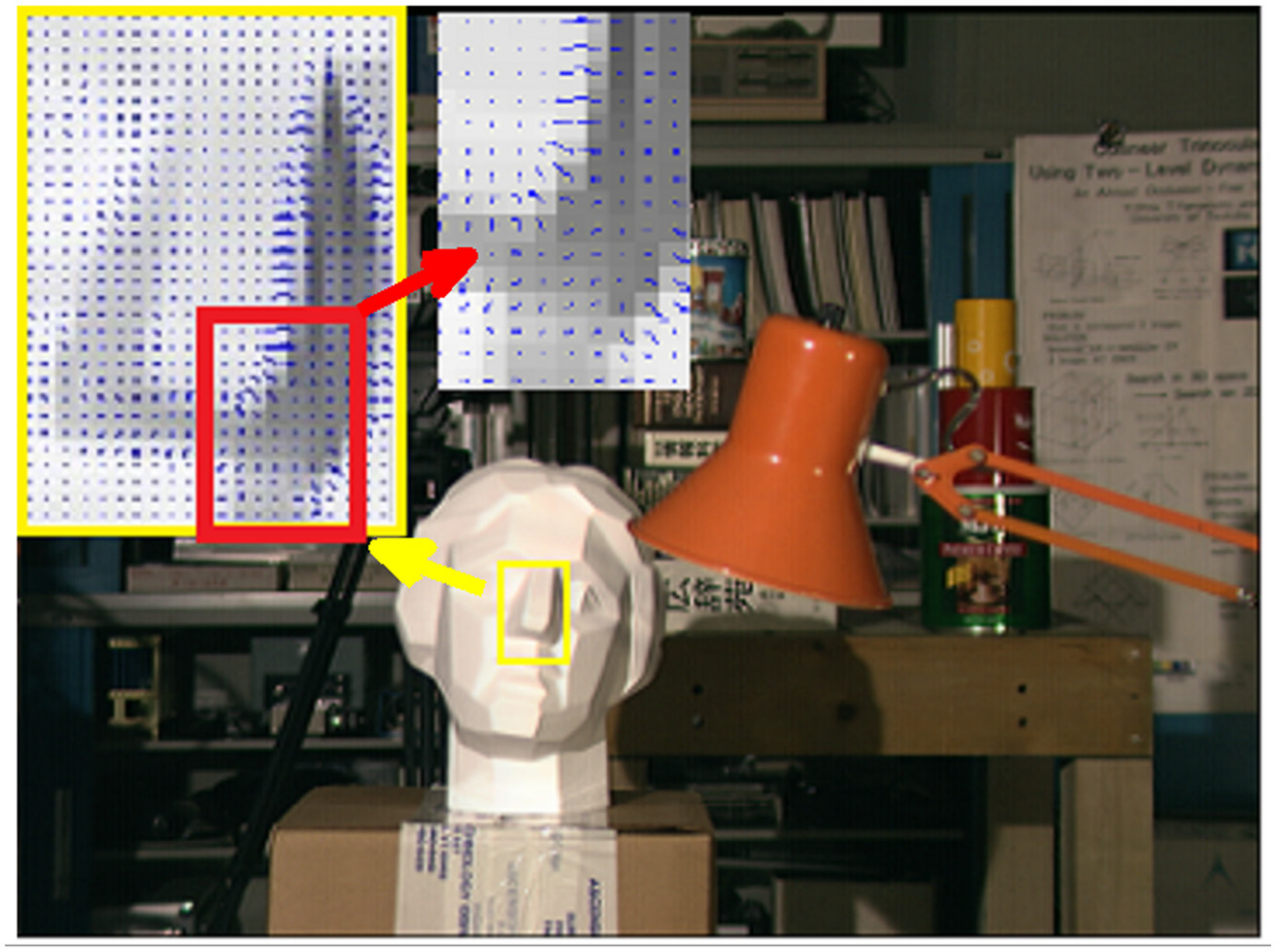
Gradient images on the Middlebury dataset “Tsukuba”. We reconstruct the gradient image based on gradient measure and zoom in on the nose region marked by the yellow rectangle. We also zoom in on the discontinuous region near the nose region marked by the red rectangle. Note that adding a gradient measure can enhance the reliability of the correspondences.

To emphasize the performance of SDDT, additional gradient measure maps for the “Tsukuba” dataset are depicted in Fig 2. For better visualization, we show a zoomed-in view of the gradient map near the nose with the yellow rectangle, where the normal view follows the nose change. Again, we zoom in on a discontinuous region near the nose region marked by the red rectangle. As seen from Fig 2, the additional gradient-based measure can be helpful to reduce the errors because it applies space contextual constraint information to acquire more abundant information. Hence, the SDDT, which combines intensity-based measures and gradient-based measures, is beneficial for improving the accuracy of BP.

#### Experiments

**Fig 3:**
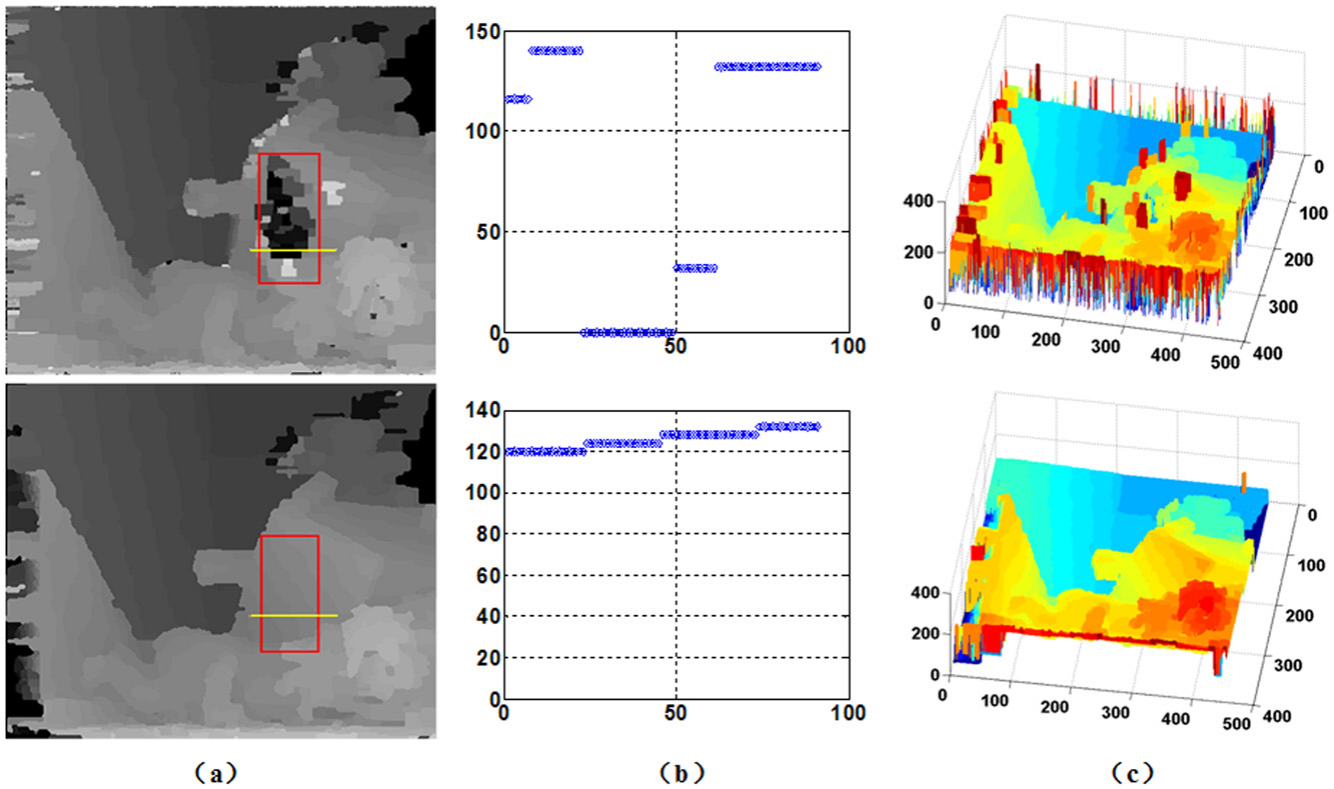
Comparison of disparity maps without and with gradient measure on “Teddy”. (a) Disparity map without and with gradient. (b) The disparity samples from (a) marked by a red rectangle. (c) 3D views based on the disparity of (a). Note that performance of flat in textureless regions with gradient measure is much better than without the gradient measure in the red rectangle.

To more intuitively contrast our SDDT and the traditional data term, we compare the two methods using the same MRF and BP optimization framework in detail using the “Teddy” datasets with abundant information in Fig 3. As shown from Fig 3, the performance of SDDT is better than the traditional data term, especially near disparity discontinuities and in textureless regions. In the red rectangle in Fig 3a, the disparity reference of the roof by the traditional data term is especially discontinuous. The primary reason is that this region is textureless, and the intensity value is nearly equal. Thus, when only using the intensity measure, the data term is not sufficiently accurate due to lacking other active constraints. Note that the gradient measure may provide auxiliary contextual inference; thus, SDDT can use space information contextual constraint. In Fig 3b, we verify our algorithm from the contour map, and SDDT is more stable and continuous. Fig 3c provides a visual 3D comparison using BP with and without SDDT. After introducing a gradient measure, the discrete effect is removed on the roof, as shown in Fig 3c. In total, the combined intensity- and gradient-based measure SDDT is more robust and accurate.

### Initial-Value Belief Propagation

As described previously, BP has indistinct edges and a slow convergence speed. To solve this problem, we present an IVBP algorithm.

#### Initial-Value Belief Propagation

Several BP algorithms have been proposed, including LBP [7], HBP [9], CBP [10], and fast-converging belief propagation [1], but they are designed to decrease time consumption without essentially improving precision. To improve precision, some researchers have combined other image or mathematics algorithms into BP, such as differential geometry [17,18,19], genetic programming [20], and image segmentation [1,21,22]. Although the accuracy of the disparity map is improved, additional methods also increase time consumption and, more importantly, do not improve the accuracy of BP itself. To address this problem, we present an IVBP to improve both accuracy and convergence speed of BP.

At present, the convergence of BP has attracted much attention [23]. However, whether a BP algorithm is convergent and what condition ensures convergence cannot be strictly determined theoretically, which becomes a limitation for BP. Even if the BP is convergent, the convergence speed and accuracy are not guaranteed in most cases. Fortunately, we know that a more accurate initial value is helpful for acquiring both a more accurate solution and faster convergence, which solves the limitation to a certain degree.

To demonstrate the advantages of IVBP, we utilize a “up-down-left-right” accelerated BP, which is a fundamental and fast BP [7]. The alternative schedule is to propagate messages in one direction (the up, down, left, and right directions) and update each node immediately.

#### Setting the Initial Value

The accuracy and time consumption of methods acquiring initial values are important for BP. Numerous local methods have been presented, such as AD, Birchfield and Tomasi dissimilarity (BT)[24], non-parametric transforms[25,26], geodesic filter [27] and BF [12,28]. The key limitations of these methods are the compatibility of both accuracy and convergence speed. At present, GF offers high speed and accurate performance and is one of the best local methods [13, 14]. Here, we provide a brief description.

We consider GF as a general linear filtering problem. Given a pair of images ϕ = {*I*,*I*′}, where *I* and *I*′ are the left and right images, respectively, a pixel *s* in *I* may match the corresponding pixel *s*′ in *I*′ with the disparity *d*_*s*_. Let *C*(*s*,*d*_*s*_) denote the cost function for pixel *s* at disparity *d*_*s*_. Cost measures *C*(·) are defined as follows [13, 14]:

$$C(s,d_{s})=(1-\lambda )\cdot \text{min}(\|I_{s+d_{s}}-{I^{\prime }}_{s}\|,\tau_{c})+\lambda \cdot \text{min}(\|\nabla_{X}I_{s+d_{s}}-\nabla_{X}{I^{\prime }}_{s}\|,\tau_{g}),\forall \lambda \in [0,1].$$ (8)

Here, ∇_*X*_ denotes the forward gradient to the right in the *X* direction, the normalization coefficient *λ* balances the color and gradient measures, and *τ*_*c*_ and *τ*_*g*_ are the truncation thresholds for the color and gradient measures, respectively.

Furthermore, the cost measure *C*_*G*_(·) of GF is output as follows:

$$C_{G}(s,d_{s})=\underset{j\in \omega_{k}}{\sum }W_{sj}(I)C(s,d_{s}),$$ (9)

Where $W_{sj}(I)=\frac{1}{{\left|\omega_{k}\right|}^{2}}\underset{(s,j)\in \omega_{k}}{\sum }\left(1+{\left(I_{s}-\mu_{k}\right)}^{T}{\left(\underset{k}{\sum }+\varepsilon U\right)}^{-1}(I_{s}-\mu_{k})\right)$. Here, *μ*_*k*_ and $\sigma_{k}^{2}$ are the mean and variance in the squared windows *ω*_*k*_ centered at pixel *k*.

Once the cost volume slices are filtered, the Winner-Takes-All (WTA) strategy is applied to select the best disparity value $d_{s}^{*}$ for each pixel *s*:

$$d_{s}^{*}=\underset{d_{s}\in \sigma }{\text{argmin}}C_{G}(s,d_{s}).$$ (10)

#### The Initial Value Using GF

To describe our IVBP, we first review how message inference is formulated. We utilize the max-product algorithm to obtain the Maximum A Posteriori (MAP) estimate of the disparity map.

The max-product BP algorithm works by passing messages around the graph, which is commonly defined by a four-connected image grid. Let $m_{p\to q}^{i}(f_{p},f_{q})$ be the message vector passed from node *p* with disparity *f*_*p*_ to one of its neighbors *q* with *f*_*q*_ at time *i*. Let *N*(*p*) \ *p* denote the set of nodes neighboring *p* other than *p* itself. Then, the iterative message passing procedure of the max-product BP algorithm is given as follows:

$$m_{p\to q}^{i+1}(f_{q})=\underset{f_{p}}{\text{max}}\left[\psi_{pq}(f_{p},f_{q})m_{p}^{i}(f_{p})\overset{}{\underset{s\in N(p)\backslash p}{\prod }}m_{s\to p}^{i}(f_{p})\right].$$ (11)

**Fig 4:**
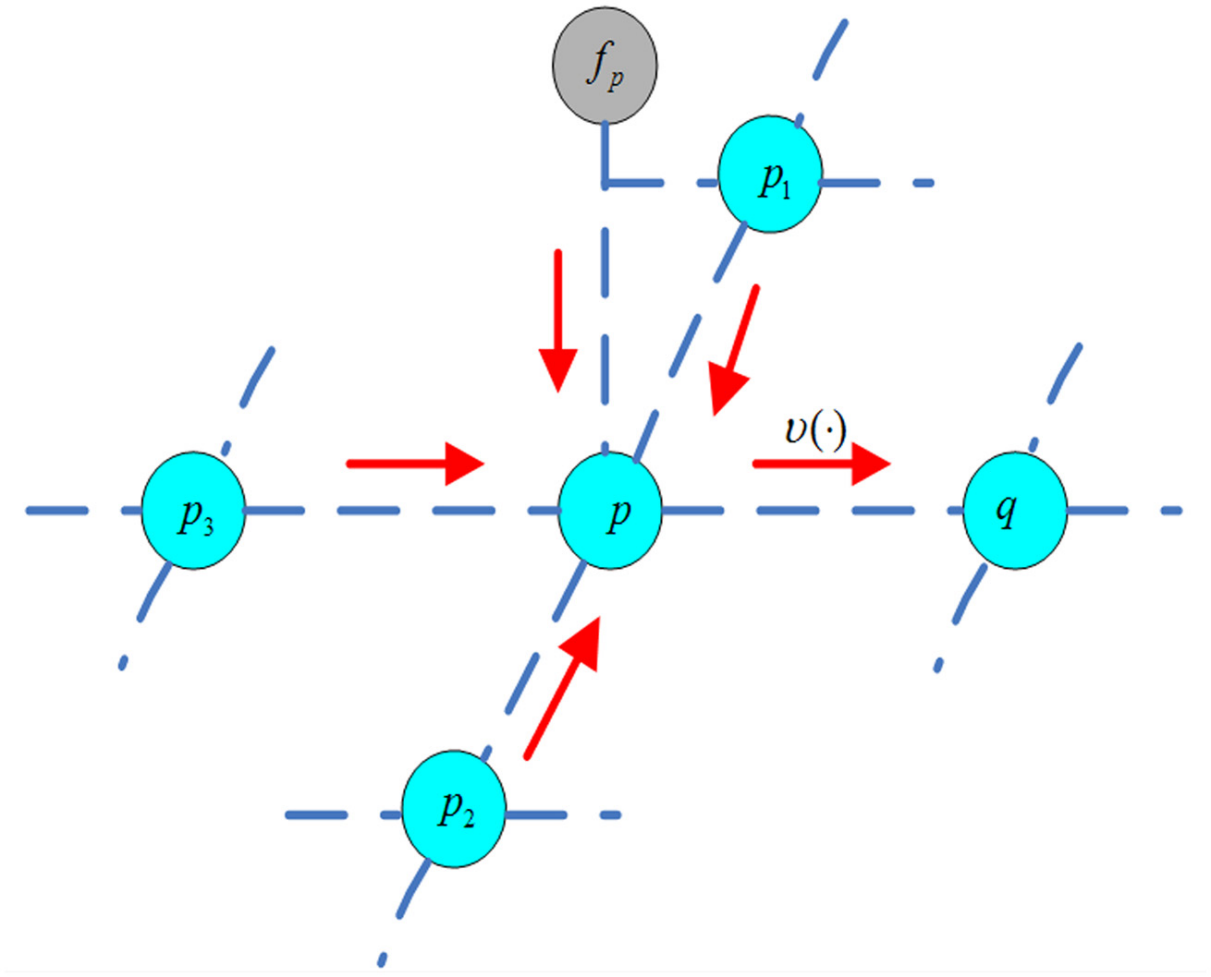
Local message passing procedure in a Markov network. Green nodes are intensive variables, where gray nodes are observable variables. The new message sent from node *p* to *q* is computed with probability *υ*(·).

Note that passing message *m*(·) represents a probability, which often determines the results of the disparity map. Generally, traditional belief vector *b*_*p*_ at *p* with *L* dimensions contains possible labels *b*_*p*_ = [*b*_*p1*_,*b*_*p2*_,…,*b*_*pL*_] with equal probabilities *P* = [*P*_*1*_,*P*_*2*_,…,*P*_*L*_], where *P*_*i*_ = *P*_*j*_ (∀*i*,*j* ∈ *L*); however, there are different probabilities due to the existence of the truth disparity value. Based on the above analysis, we set the results of GF as the initial value of the BP algorithm and different probabilities *P*_*i*_ in different disparity values *b*_*i*_ using the piecewise probability function *υ*(·). That is, disparities derived from GF have greater probabilities than other disparity values because their disparities are much closer to the true disparity values, as shown in Fig 4. To avoid possible errors, we set the disparity value $d_{p}^{*}$ of GF at node *p* as the higher probability weight *α*_1_ and set the lower probability weight *α*_2_ near the disparity value $d_{p}^{*}$ of GF. Hence, the piecewise function *υ*(·) is defined as follows:

$$\upsilon (f_{p})=\{\begin{array}{c} \alpha_{1},\begin{array}{cc} & if\begin{array}{cc} & f_{p}=d_{p}^{*}, \end{array} \end{array}\\ \alpha_{2},\begin{array}{cc} & if\begin{array}{cc} & f_{p}\in N(d_{p}^{*}) \end{array} \end{array}\\ \begin{array}{cc} 1, & if\begin{array}{ccc} & & f_{p}\notin N(d_{p}^{*}). \end{array} \end{array} \end{array},\;where\begin{array}{cc} & \alpha_{1}>\alpha_{2} \end{array}.$$ (12)

Here, *υ*:ℜ→ℜ, the terms *α*_1_ and *α*_2_ are the probability thresholds of different messages, *α*_1_ > *α*_2_ > 1, and d* is the disparity value derived from GF, which belongs to a finite set of labels *b* = [*b*_*1*_,*b*_*2*_,…,*b*_*L*_] with *L* discrete disparities. See Section 3.1 for a discussion of the parameter settings for *α*_1_ and *α*_2_ and their sensitivities.

From Eqs (11)) and (12), improved passing message m*(*f*_*p*_) is calculated:

$$m^{*}(f_{p})=m(f_{p})⊗\upsilon (f_{p}).$$ (13)

For simplification, we only analyze the data term and do not consider different messages in the up, down, right and left directions. Inserting Eq (13) into Eq (11), the improved iterative message passing procedure is given as follows:

$$m_{p\to q}^{*(i+1)}(f_{q})=\underset{f_{p}}{\text{max}}\left[\psi_{pq}(f_{p},f_{q})m^{*}{}_{p}^{i}(f_{p})\overset{}{\underset{s\in N(p)\backslash p}{\prod }}m^{*}{}_{s\to p}^{i}(f_{p})\right].$$ (14)

Further, the improved belief vector $b_{p}^{*}$ at node *p* can be computed after iteration *i*:

$$b_{p}^{*}(f_{p})=m_{p}^{*}(f_{p})\underset{p\in N(f_{p})}{\prod }m_{p\to q}^{*}(f_{p}).$$ (15)

Finally, the label $f_{p}^{*}$ maximizing $b_{p}^{*}(f_{p})$ at node *p* is selected as follows:

$$f_{p}^{*}=\underset{p\in P}{\text{argmax}}b_{p}^{*}(f_{p}).$$ (16)

Note that our IVBP contains BP as a special case, when both *α*_1_ = 1 and *α*_2_ = 1.

The disparity value of GF is used as the constraint of the smooth term and to assist as an information inference. Thus, IVBP has good performance, as shown by the experimental results in Section 3.

#### Post-processing

Aim of our algorithm is essentially improving the BP, but our methods can also combine very well with other methods, such as image segmentation.

There are some shortcomings in BP, such as the MRF shrinking bias, over-smoothness phenomenon, and ambiguous edges of disparities. Image segmentation can solve these problems, which may decompose the reference image into a series of homogeneous color or grayscale regions. Image segmentation can help resolve ambiguity within textureless regions and enable object boundaries corresponding to depth discontinuities. Therefore, IVBP with image segmentation (denoted as WIVBP) is proposed. See Section 3.1 for the discussion of WIVBP.

## Experiments and Discussions

In this section, we conduct experiments to evaluate our algorithm with some quantitative error analysis. First, we analyze the key parameter settings of the proposed algorithm and then perform experiments on the Middlebury and new 2014 Middlebury datasets with respect to accuracy and convergence speed. The experimental results show the advantages of accuracy and efficiency for our algorithm compared with other BP algorithms.

### Parameter Settings

Here, we provide some of the important parameter settings used in our algorithm, including the optimal weight $\varpi^{\prime }$ and the probability thresholds of different messages *α*_1_ and *α*_2_.

**Fig 5:**
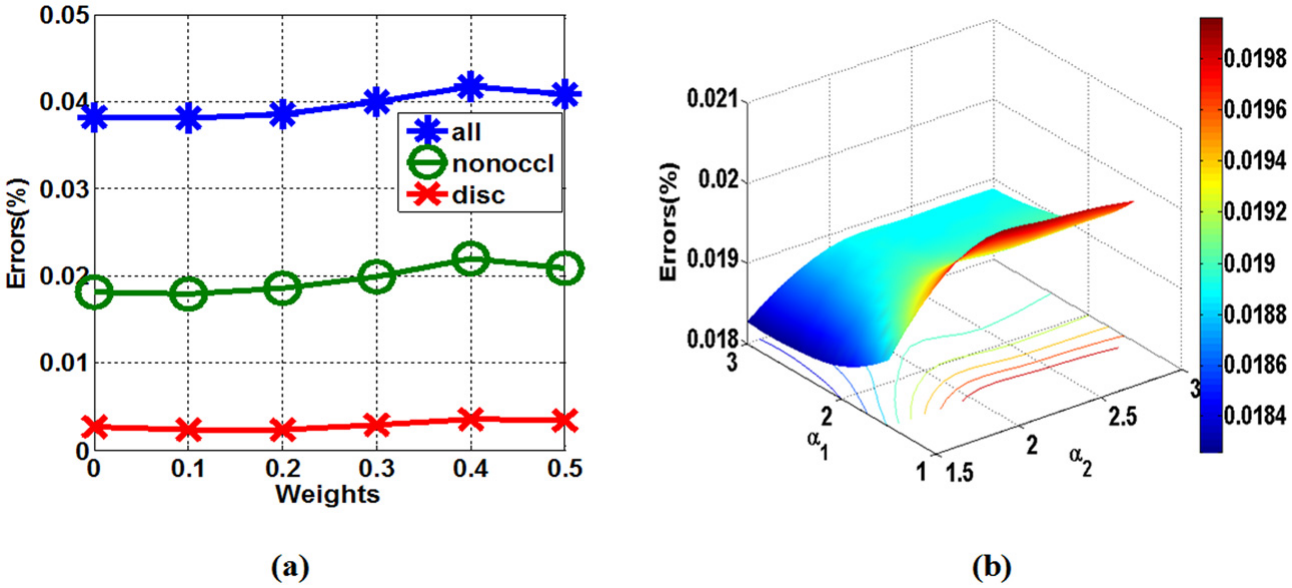
(a) Performance of the optimal weighting $\varpi^{\prime }$ from 0 to 0.5 between the intensity measures and gradient measures on the Middlebury “Tsukuba” dataset. (b) Performance of *a*_1_ and *a*_2_ on the Middlebury “Tsukuba” dataset.

For SDDT, the parameter $\varpi^{\prime }$ is the optimal weighting between intensity- and gradient-based measures. Fig 5a shows the performance of the proposed algorithm according to different optimal weighting $\varpi^{\prime }$ from 0 to 0.5 in the “Tsukuba” dataset. Fig 5 indicates that $\varpi^{\prime }$ = 0.1 is relatively better within the range of 0 and 0.5. When $\varpi^{\prime }$ = 0.1, the percentage of badly matched pixels is lowest because images blur when $\varpi^{\prime }$ is relatively larger.

We also evaluate the probability thresholds of different messages *α*_1_ and *α*_2_ through the errors of non-occluded regions, as shown in Fig 5b, which appears fairly robust and stable in the range of *α*_1_ ∈ [1.5,3] and *α*_2_ ∈ [1.5,3], with the error range of 0.0183 to 0.0198. As seen from Fig 5b, the lowest value occurs when *α*_1_ = 2 and *α*_1_ = 1.5.

### Experimental Results

We evaluate our proposed algorithm with the Middlebury and new 2014 Middlebury datasets [4]. First, the overall performance of our algorithm is depicted with the Middlebury datasets. Simultaneously, our algorithm is compared with other excellent BP algorithms. Then, we also test our algorithm on the Middlebury and new 2014 Middlebury datasets.

#### Measure Settings

To conveniently evaluate the performance of our algorithm, we provide an abbreviation of the six different measures according to the percentages of ‘bad’ pixels [4], as shown in Table 1:

**Table 1:** Abbreviations of six different measures.

Measure	all	non-occluded	near occluded	occluded	textured	textureless
Abbreviation	all.	non.	neocc.	occ.	tex.	untex.

#### Performance on the Middlebury data set

**A. Total Performance.** Generally, we evaluate the performance of disparity maps using the percentages of ‘bad’ pixels, which are primarily among “non.”, “all.” and “disc.”. Table 2 depicts the performance of our proposed algorithm by reporting a comparison with other state-of-the-art BP methods on the Middlebury datasets “Tsukuba”, “Venus”, “Teddy” and “Cones” with an error threshold of 1. It can be seen that our WIVBP (IVBP combining image segmentation) can achieve the better accuracy than other state-of-the-art BP methods and even local methods [29,30], demonstrating the competitiveness of our proposed method. Meanwhile, our IVBP can intrinsically improve the accuracy of BP. Table 2 shows that IVBP outperforms the other BP algorithms [25,31,32,33,34,35,36,37] except TSGO [38] with the lowest average errors. Note that the errors by our IVBP are greatly reduced, compared with other BP except TSGO. At present, TSGO is best BP algorithm listed in the Middlebury datasets. However, TSGO is obtained through four post-processing methods, which greatly decease the average error from 5.70% to 4.06% (see [38]). Relatively, our IVBP does not use post-processing steps and better than TSGO without post-processing methods. Our IVBP is one of the best BP algorithms at present.

**Table 2:** Comparison of the results with an error threshold of 1 in the Middlebury dataset. Our algorithms (IVBP and WIVBP) are shown.

		Middlebury											
Algorithm	Mean Error (%)	Tsukuba (%)			Venus (%)			Teddy (%)			Cones (%)		
		non	all	disc	non.	all	disc	non.	all	disc	non	all	disc
***WIVBP***	**5.16**	**1.30**	**1.82**	**5.37**	**0.24**	**0.70**	**3.14**	**5.50**	**10.9**	**12.4**	**3.12**	**9.55**	**7.89**
*TSGO[38]*	4.06	0.87	1.13	4.66	0.11	0.24	1.47	5.61	8.09	13.8	1.67	6.16	4.95
*HistoAqqr2[29]*	5.20	1.93	2.30	6.39	0.16	0.46	2.22	5.88	11.3	14.7	2.41	7.78	6.89
***IVBP***	**5.68**	**1.83**	**3.13**	**6.91**	**0.33**	**1.10**	**3.82**	**5.58**	**11.6**	**13.2**	**3.07**	**9.97**	**7.72**
*TSGO* ^*]*^ *[38]*	5.70	0.93	1.28	4.93	0.88	1.49	9.71	6.24	9.40	15.7	2.77	7.66	7.45
PlaneFitBP[31]	5.78	0.97	1.83	5.26	0.17	0.51	1.71	6.65	12.1	14.7	4.17	10.7	10.6
SCoBeP[32]	5.90	1.47	2.01	7.92	0.24	0.62	3.28	6.22	11.7	15.7	3.49	8.84	9.32
SymBp+occ[33]	5.92	0.97	1.75	5.09	0.16	0.33	2.19	6.47	10.7	17.0	4.79	10.7	10.9
OverSegmBP [34]	6.11	1.69	1.97	8.47	0.51	0.68	4.69	6.74	11.9	15.8	3.19	8.81	8.89
RTAdapt Wqt[30]	6.20	1.45	1.99	7.59	0.40	0.81	3.38	7.65	13.3	16.2	3.48	9.34	8.81
MVSegBP [25]	6.34	1.06	2.78	5.57	0.20	0.61	2.02	6.53	11.3	14.8	5.29	11.3	14.5
EnhancedBP [35]	6.69	0.94	1.74	5.05	0.35	0.86	4.34	8.11	13.3	18.5	5.09	11.1	11.0
RealtimeBP [36]	7.69	1.49	3.40	7.87	0.77	1.90	9.00	8.72	13.2	17.2	4.61	11.6	12.4
CSBP [37]	11.4	2.00	4.17	10.5	1.48	3.11	17.7	11.1	20.2	27.5	5.98	16.5	16.0

1. Do not include post-processing methods.

**Fig 6:**
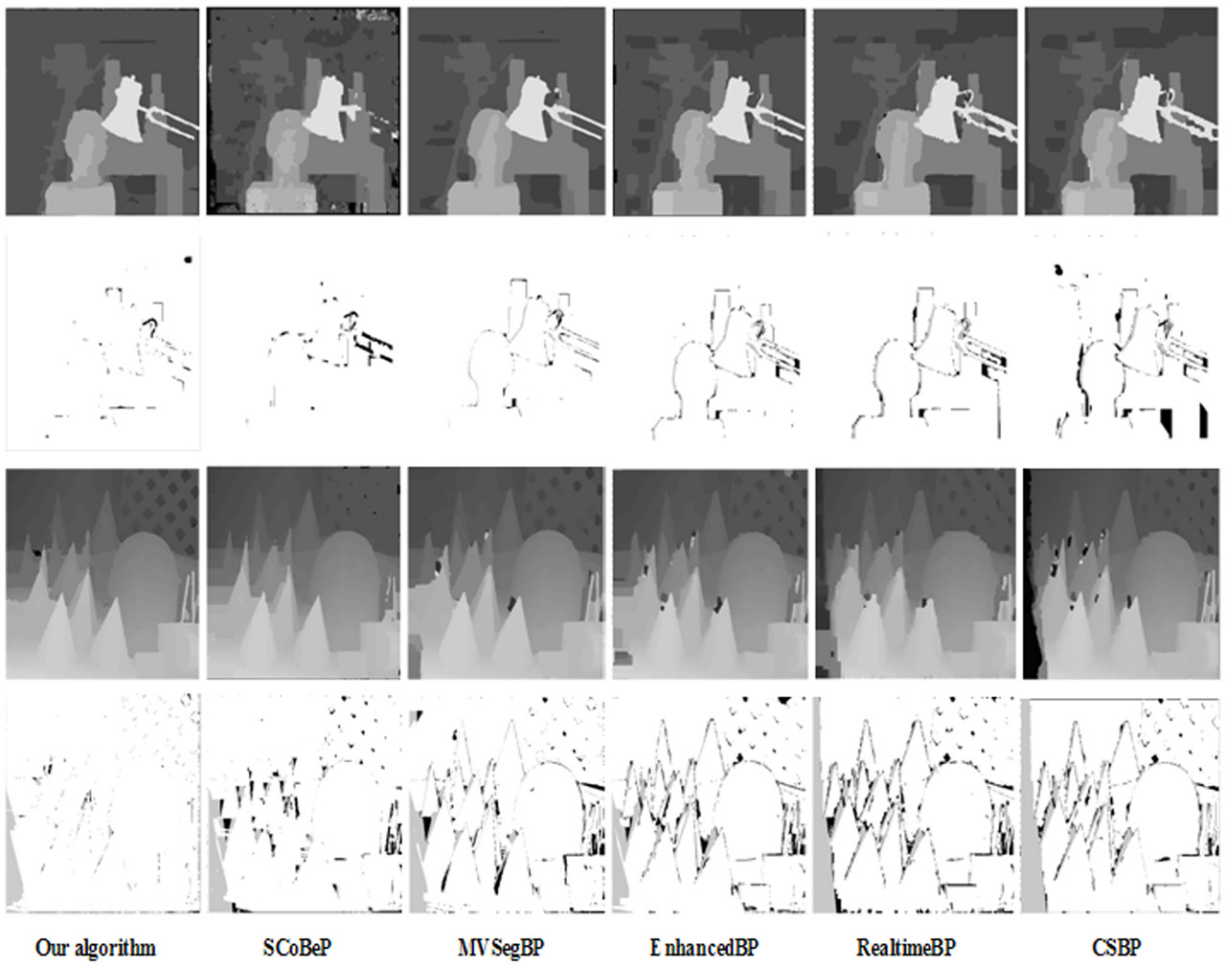
Comparison of our algorithm and five other BP algorithms with the Middlebury datasets “Tsukuba” and “Cones”, respectively. Note that our algorithm has much better edge-preserving smooth effects compared with the other BP algorithms

Our algorithm also has better edge-preserving smooth effects compared with the other excellent BP methods, especially near disparity discontinuities and in textureless regions. For simplicity, we show the results of five BP methods only, as shown in Fig 6. The reason our method has better performance is because the inference of the BP algorithm with SDDT results in more accurate performance, and simultaneously an accurate initial value can be helpful in acquiring better accuracy.

**B. Analysis of Accuracy Improvements.** The addition of different methods can improve the accuracy of BP, as shown in Table 3. In Table 3, the results of our SDDT, IVBP and WIVBP are shown, which show the contribution of our SDDT and IVBP. It can be seen that SDDT and IVBP are crucial to obtain excellent BP results.

**Table 3:** Comparison of the results with an error threshold of 1 in the Middlebury dataset. Our algorithms BP with SDDT (denoted BP-SDDT), IVBP and WIVBP are shown.

		Middlebury											
Algorithm	Mean Error (%)	Tsukuba (%)			Venus (%)			Teddy (%)			Cones (%)		
		non	all	disc	non.	all	disc	non.	all	disc	non	all	disc
*WIVBP*	5.16	1.30	1.82	5.37	0.24	0.70	3.14	5.50	10.9	12.4	3.12	9.55	7.89
*IVBP*	5.68	1.83	3.13	6.91	0.33	1.10	3.82	5.58	11.6	13.2	3.07	9.97	7.72
*BP-SDDT*	9.63	3.66	4.65	10.5	1.51	2.39	18.2	9.14	15.9	20.3	4.84	12.1	12.4
*BP*	13.8	4.28	6.29	13.2	3.03	3.36	23.0	16.3	22.6	26.1	10.6	18.1	19.9

**Fig 7:**
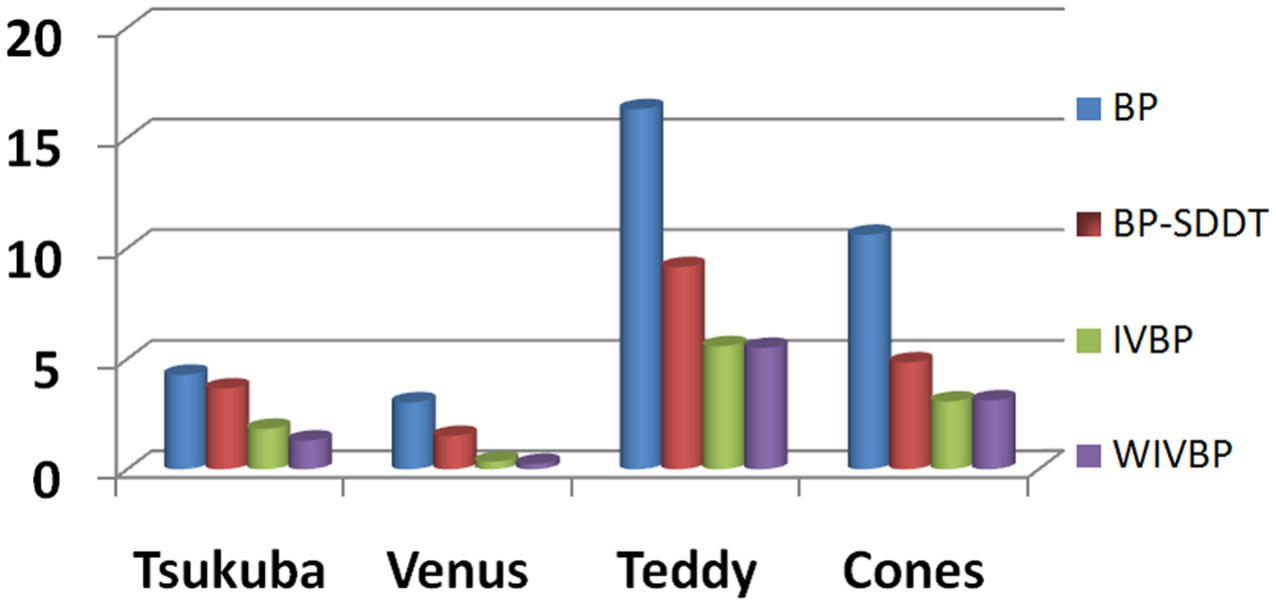
Comparison diagram of the errors using four methods (BP, BP-SDDT, IVBP, and WIVBP) in non-occluded measures (non.) in the Middlebury datasets “Tsukuba”, “Teddy”, “Cones” and “Venus”.

**Fig 8:**
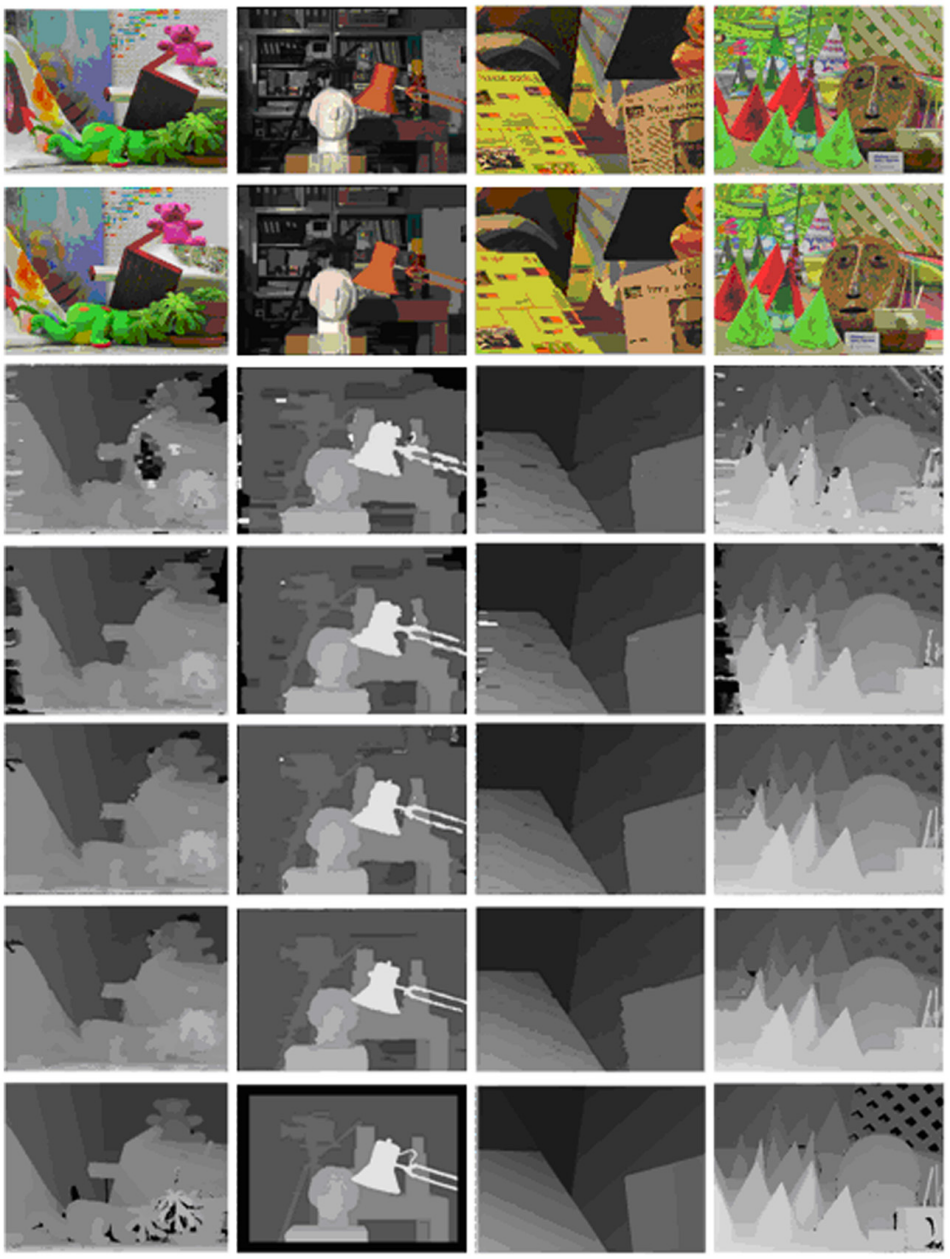
Performance on the Middlebury datasets using four methods (BP, BP-SDDT, IVBP, and WIVBP). From left to right: “Teddy”, “Tsukuba”, “Venus” and “Cones”. From top to bottom: left reference images, image segmentation maps, disparity maps of BP, BP-SDDT, IVBP, and WIVBP, and the ground truth disparity maps.

For comparison, we provide a visual comparison of the progressive improvement process for the four proposed methods, namely BP, BP-SDDT, IVBP and WIVBP. Fig 7 shows the. comparison diagram of the errors using four methods (BP, BP-SDDT, IVBP, and WIVBP) in non-occluded measures in the Middlebury datasets “Tsukuba”, “Teddy”, “Cones” and “Venus”, and Fig 8 shows that the each progressive improvement is clear, especially in the textureless and occluded regions. Specifically, relative to only the intensity measure, BP-SDDT performs better, possibly because SDDT can imply gradient measures (contextual constraint information) to effectively improve accurate judgments. For IVBP, an accurate initial value is helpful to promote the probability of information propagation.

**Fig 9:**
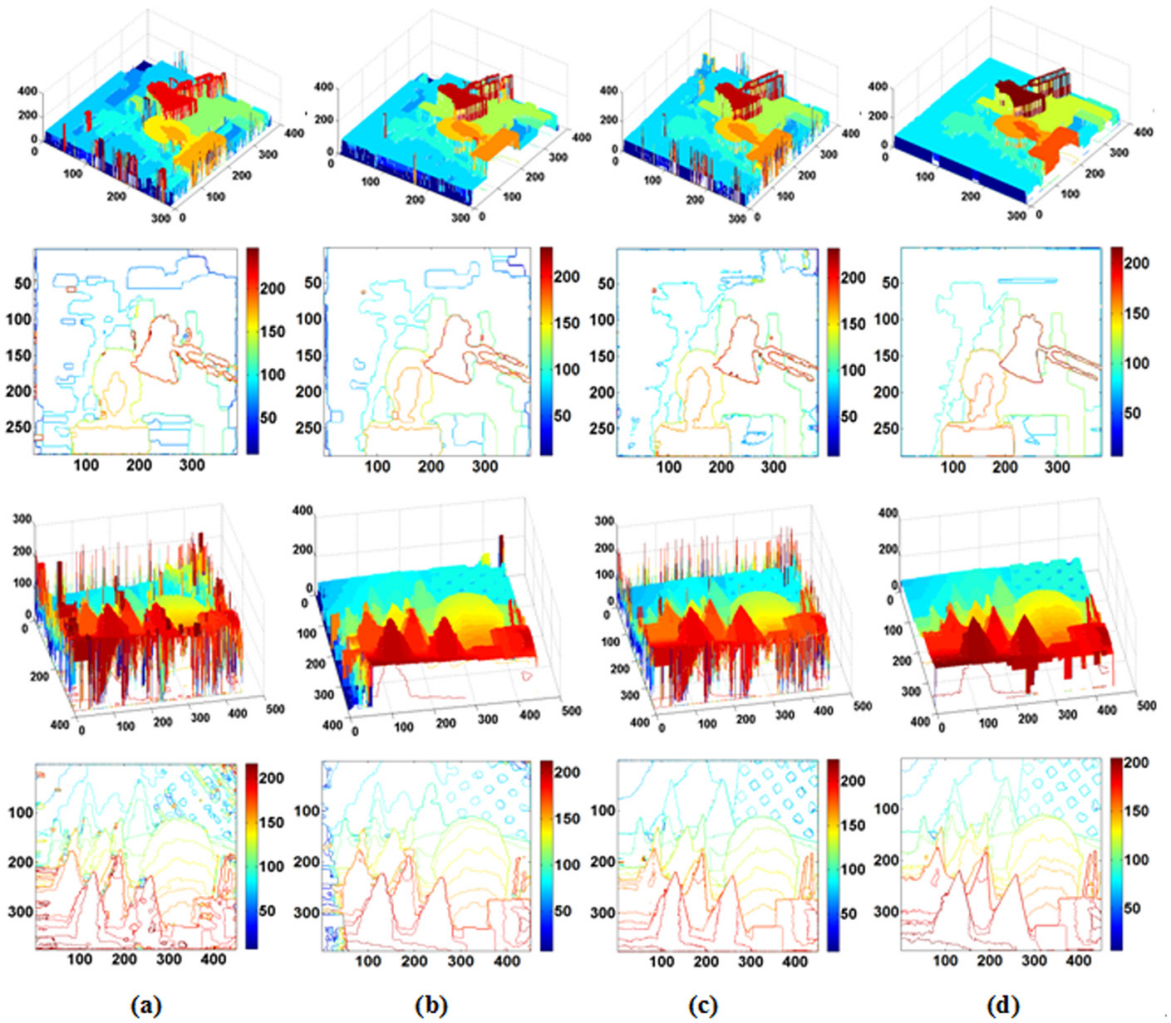
Comparison of our algorithms by 3D and contours on the Middlebury datasets “Cones” and “Tsukuba”. (a) BP, (b) BP-SDDT, (c) IVBP, and (d) WIVBP.

For more intuitive comparisons, we rebuild 3D scenes and their contour maps based on the disparity maps of the previous four methods (BP, BP-SDDT, IVBP, and WIVBP) for “Tsukuba” and “Cones” in Fig 9. IVBP and WIVBP have better 3D effects and accurate contours, especially in the textureless and discontinuous regions.

**Fig 10:**
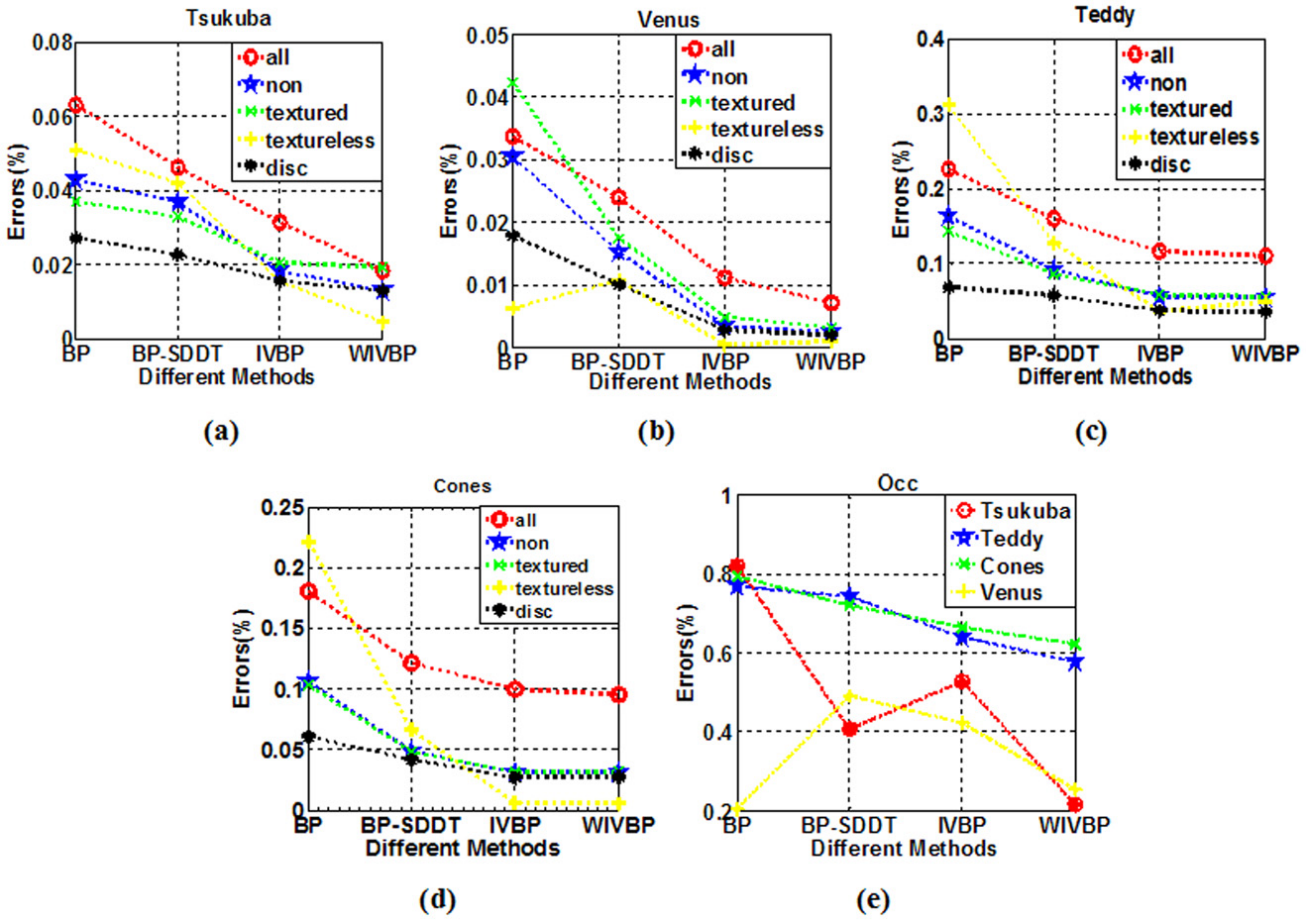
Error statistics for the percentage of ‘bad’ matching pixels at six different thresholds. (a) “Tsukuba”, (b) “Teddy”, (c) “Venus”, and (d) “Cones”, including all pixels, pixels in non-occluded areas, pixels in textured areas, pixels in textureless areas and pixels in discontinuous areas. (e) Error statistics for the four methods in the occluded areas.

We evaluate our algorithms on all six measures listed in Table 1. Fig 10 shows that our algorithm outperforms the traditional BP algorithms on six different measures for “Tsukuba”, “Venus” and “Cones” but not for “Teddy”. Because the occluded measure errors are relatively high, to avoid the influence of other measures, we independently depict them in Fig 10e. In Fig 10e, our algorithms (BP-SDDT, IVBP, and WIVBP) are nearly lower than BP, especially for WIVBP. Occlusion is often caused by the loss of the corresponding pixels in left or right views. We may assume that the color value of the occluded pixels is similar to the neighboring pixels. Therefore, the results of the image segmentation in the left views can help judge the disparity values in the occluded regions.

### Performance for Other Images

Here, we analyze our algorithm on more challenging Middlebury and 2014 new Middlebury datasets and simultaneously compare results with some excellent BP algorithms, such as HBP [9], Real-time Hierarchical Belief Propagation (Realtime BP)[36], Constant-Space Belief Propagation (CSBP)[37], Two Step Global Optimization (TSGO)[38],etc.

**Fig 11:**
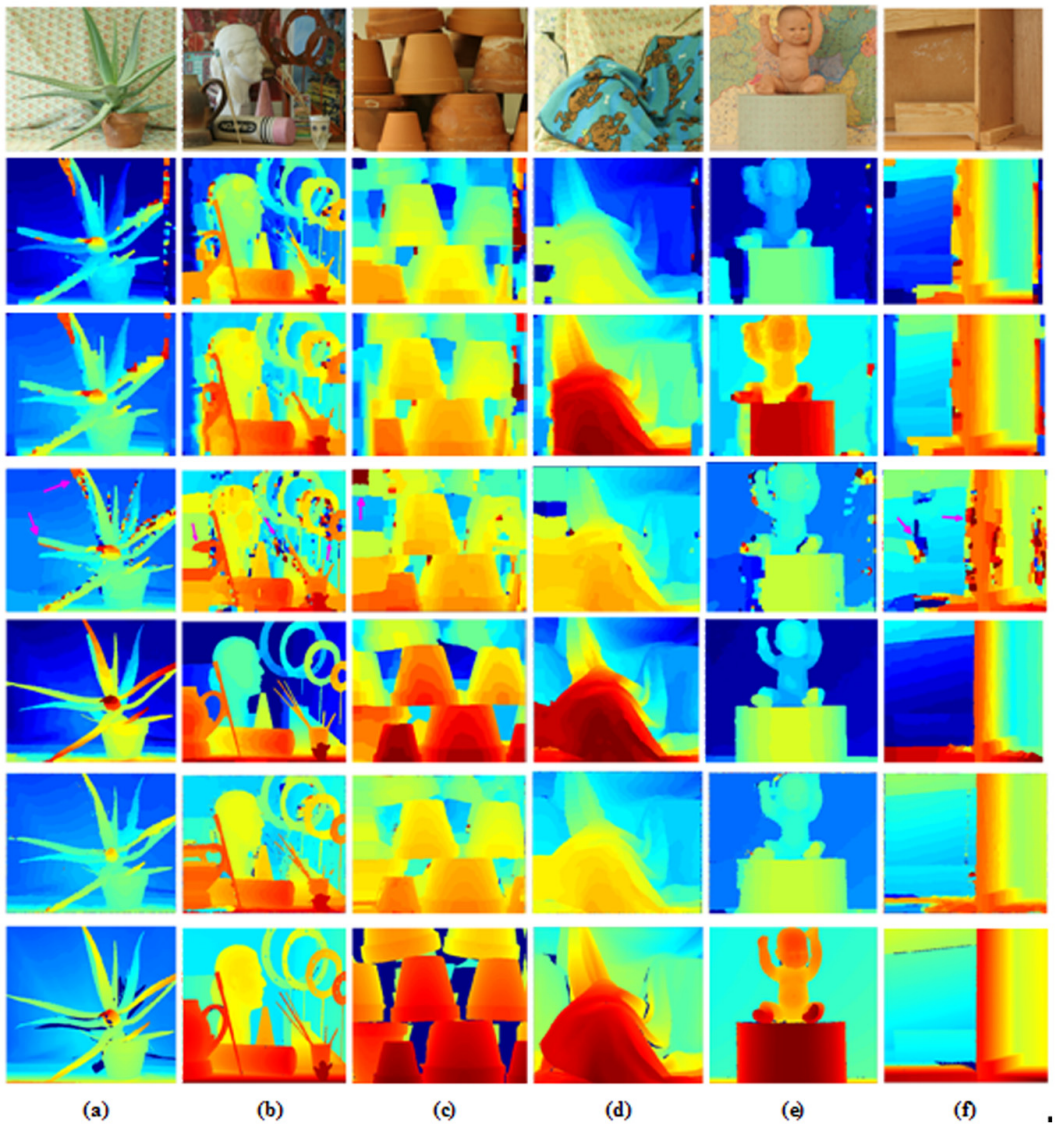
Results on the Middlebury datasets. (a) “Aloe”, (b) “Art”, (c) “Flowerpot”, (d) “Cloth3”, (e) “Baby1”, and (f) “Wood1”. From top to bottom: disparity maps of RealtimeBP, CSBP, HBP, TSGO, IVBP and the ground truth disparity maps. Edge-preserving smoothing effects are indicated by pink arrows in our algorithm.

In Fig 11, our algorithm holds more distinct edges, decreases ambiguous disparities and acquires smoother low-texture effects on the Middlebury datasets from 2001, 2003, 2005, and 2006 compared with four excellent BP algorithms. We stress that the performance of our algorithm is much closer to the ground truth disparity maps than other BP algorithms. In Table 4, errors in the six datasets “Aloe”, “Art”, “Flowerpot”, “Cloth3”, “Baby1” and “Wood1” are shown. Note that the errors of our IVBP are greatly reduced, compared with other BP except TSGO. Note that TSGO is obtained through four post-processing methods, which greatly reduces errors. Relatively, our IVBP does not use post-processing steps, and is competitively with TSGO.

**Table 4:** Results with the Middlebury datasets “Aloe”, “Art”, “Baby1”, “Wood”, “Flowerpot”, and “Cloth3”. The errors by IVBP are greatly reduced compared with other BP algorithms.

	Aloe		Art		Baby1		Wood		Flowerpot		Cloth3	
Algorithm	non	all	non.	all	non.	all	non	all	non.	all	non	all
***IVBP***	**5.44**	**9.72**	**9.74**	**18.3**	**3.36**	**4.88**	**6.12**	**7.84**	**16.1**	**26.7**	**1.97**	**4.58**
TSGO[38]	3.81	6.28	3.46	5.06	1.27	2.05	5.49	6.19	14.5	22.3	1.42	1.36
HBP[9]	17.5	24.3	32.2	42.4	18.1	21.7	32.0	38.5	46.2	53.9	12.8	17.5
RealtimeBP[36]	45.1	47.3	19.5	31.7	43.0	49.3	46.7	51.6	63.9	68.9	43.4	40.5
CSBP[37]	42.0	47.8	19.1	30.9	43.0	45.2	46.9	49.4	66.4	69.1	45.1	48.3

**Fig 12:**
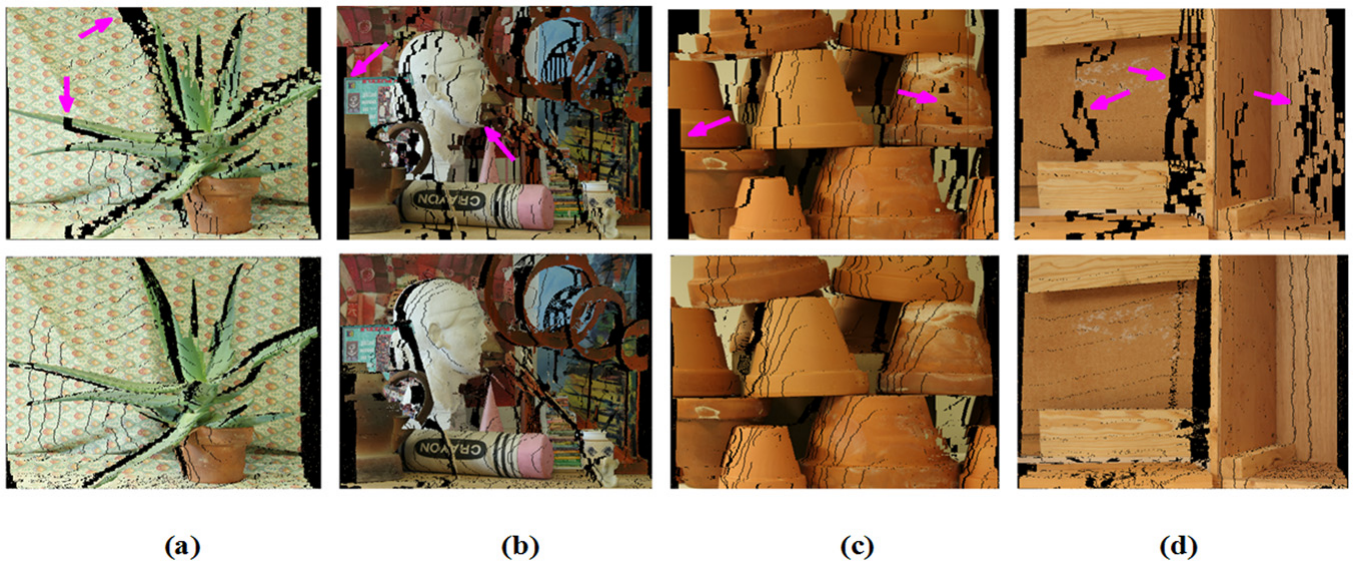
Synthesized views with HBP and IVBP from Fig 11 in the Middlebury datasets. Poorer edge-preserving smoothing properties are observed for HBP compared with IVBP, as indicated by pink arrows.

Fig 12 provides a visual comparison based on the synthesized views using the disparity maps from Fig 11. The performance improvement of our algorithm is clear. Note that our algorithm has better accuracy and edge-preserving smoothing effects compared with other BP algorithms, as shown in Figs 11 and 12.

**Fig 13:**
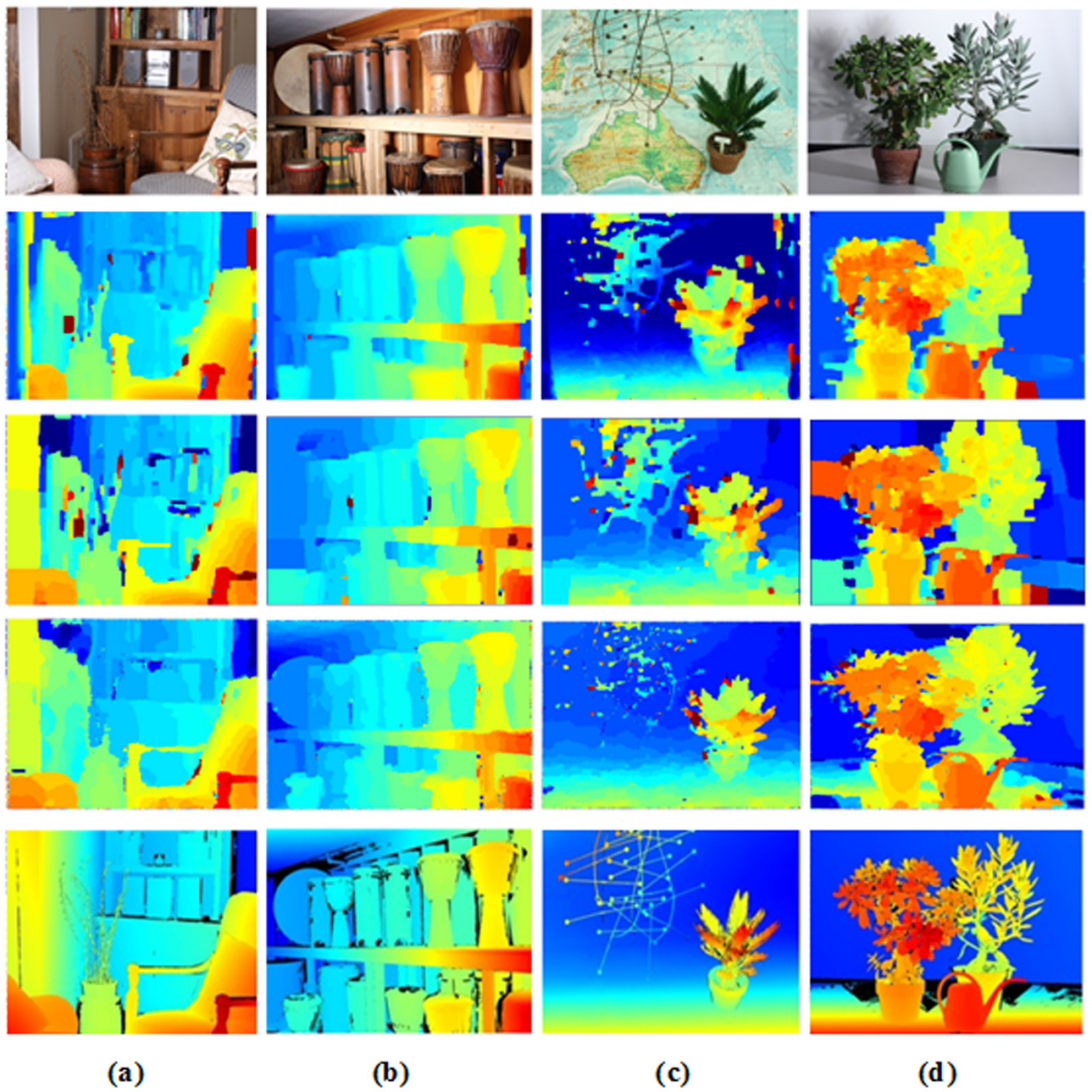
Performance with the 2014 new Middlebury datasets. (a) “Livingroom”, (b) “Djembe”, (c) “Australia”, and (d) “Plants”. From top to bottom: left reference images, disparity maps of BP algorithm, disparity maps of CSBP algorithm, disparity maps of IVBP, and the ground truth disparity maps.

Presently, the old Middlebury benchmark suffers from over-fitting. Relatively, the new 2014 Middlebury datasets consist of more challenging and realistic scenes. In Fig 13, the accuracy of IVBP is greatly improved in sophisticated scenarios compared with HBP and CSBP algorithm.

#### Running Time Performance

Our algorithm also greatly accelerates convergence speed of BP while keeping the good accuracy. Here, we analyze the improvement of the convergence speed of our algorithm in detail.

For traditional BP, a large number of iterations are required to guarantee convergence and achieve high-quality results. Relatively, our IVBP can obtain low iteration times and fast convergence speed.

Our algorithm can greatly decrease iteration times. According to (3), the energy function value *E* ^*i*^(·) is updated after iteration *i*. For every iteration *i* and *i*-1, if the L1-nrom of the relative energy errors is larger than a threshold *η*, i.e. |(*E* ^*i*^(·)−*E* ^*i-1*^(·))/*E* ^*i*^(·)| ≥ *η*,*E* ^*i*^(·) is updated, otherwise *E* ^*i*^(·) is declared as convergence. In our experiments, we set respectively *η* = 10^−4^ and *η* = 10^−5^. Table 5 shows that IVBP only needs 3 iterations when *η* = 10^−4^, whereas BP needs 8 iterations. It is obvious to greatly decrease convergence times for IVBP compared to BP.

**Table 5:** Comparison of performance of BP and IVBP in “Tsukuba” datasets.

	10^−4^		10^−5^	
	Iteration Times	Running Time(s)	Iteration Times	Running Time(s)
IVBP	3	0.546	8	1.482
BP	8	1.578	18	3.647

**Fig 14:**
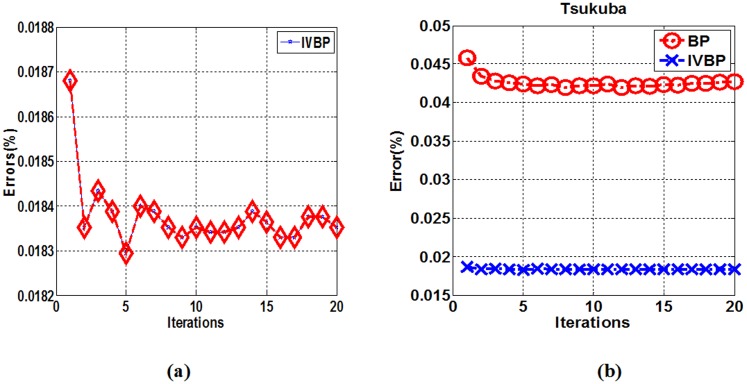
Iteration images in “Tsukuba”. (a) Iteration images with IVBP. (b) Iteration images with BP and IVBP.

Experiments demonstrate that the actual convergence speed of IVBP is drastically reduced with high accuracy. The reduction may be because a more accurate initial value requires fewer iteration times than the traditional BP algorithms to reach convergence, which is much closer to the true disparity value with much higher probability. As shown in Fig 14a, our algorithm has fast convergence speed in only a few iterations and then tends toward stability with high accuracy about 1.83%. In Fig 14b, traditional BP requires a large number of iterations and has slow convergence speed with relatively high errors about 4.2%.

Reduction of iteration times means reduction of run time. For traditional BP, the time complexity is *O*(*NDL*), with all pixels (*N*) and disparity hypotheses (D), and iteration times (L). In contrast, our IVBP can greatly reduce iteration times (*L*_*c*_ ≺ *L*) and the time complexity is *O*(*NDL*_*c*_) ≺ *O*(*NDL*). If only several iterations, the complexity is approximately thought to *O*(*ND*).

**Fig 15:**
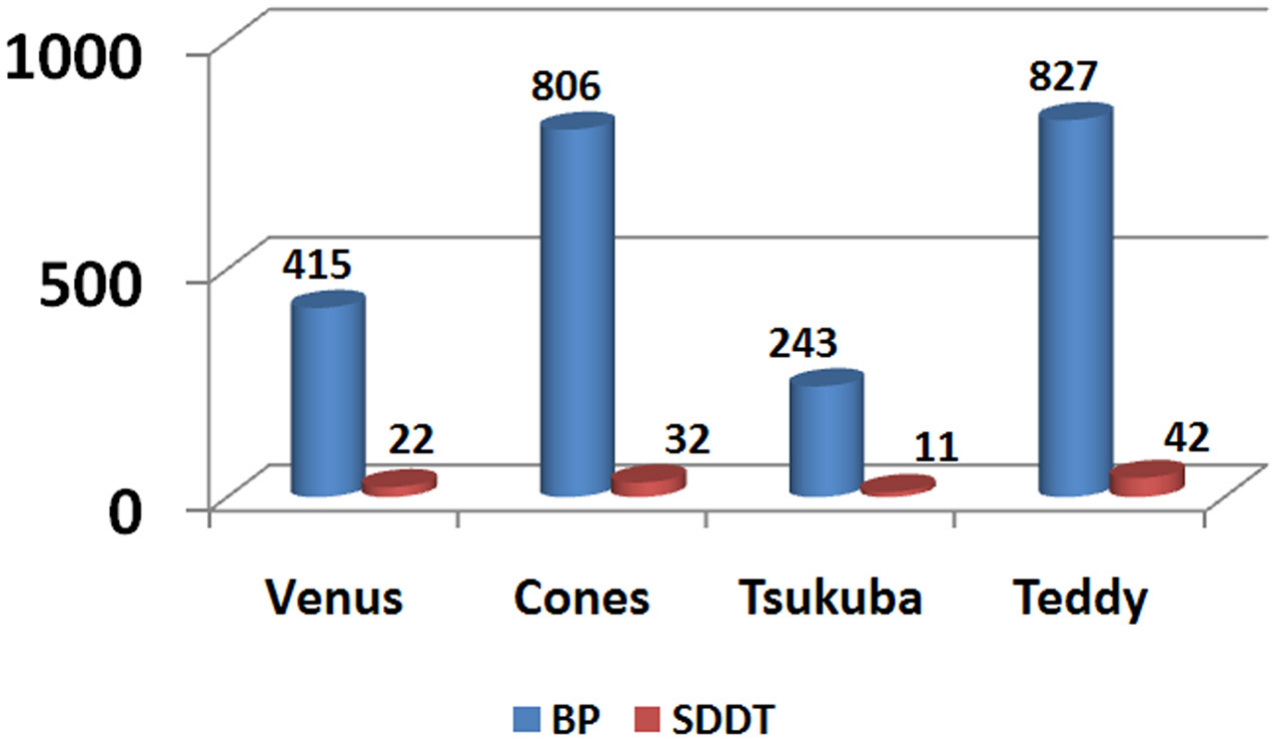
Comparison diagram of mean running time in the Middlebury datasets “Tsukuba”, “Teddy”, “Cones” and “Venus”.

To compare BP-SDDT with traditional BP in running time, we perform experiments with the Middlebury datasets “Teddy”, “Tsukuba”, “Venus” and “Cones” as examples. To avoid the influence of contingency, we run the analysis ten times and compute the mean running time. As shown in Fig 15, although SDDT is added, the additional running time is nearly negligible. According to statistics, the ratios between additional running time and traditional BP on “Tsukuba”, “Venus”, “Sawtooth”, “Cones” are 5.3%, 3.97%, 4.53%, 5.08%, respectively. Furthermore, the overall average runtime of four runtime ratios is rarely 4.72%. Compared with traditional BP, the SDDT is negligible while improving accuracy.

We experiment with a laptop with an Intel Core i3 2.37 GHz CPU and 4GB RAM. The implementation is in C++. To analyze the trade-off between complexity and accuracy, we list both the processing time and the errors in Table 6. Note that the processing time on a single-core CPU was measured for “Tsukuba” using published program[9,36,37,38] and the average error was calculated for all the test sequences. Our algorithm converge in three numbers in the accuracy of 10^−4^ using 0.546 second, while seven numbers in the accuracy of 10^−5^ using 1.482 second. Our algorithm is faster BP algorithm than other BP algorithms while holding the better accuracy. Even our method is faster than other excellent local methods.

**Table 6:** Comparison with other methods on the runtime and errors for “Tsukuba”.

Algorithm	Errors (%)	Runtime(s)
**IVBP(10** ^**−4**^ **)**	**5.69**	**0.546**
**IVBP (10** ^**−5**^ **)**	**5.68**	**1.482**
HistoAqqr2[29]	5.20	1.38
TSGO[38]^1^	4.06	3.5
RealtimeBP[36]	7.69	1.326
CSBP[37]	11.4	1.248
HBP[9]	11.2	5.0

^1^. Published program includes post-processing methods.

## Conclusions

We propose an improved BP algorithm with a novel IVBP and SDDT with regard to accuracy and convergence speed. First, we propose a novel SDDT to improve the data term of BP. Compared with intensity-based measures alone, SDDT effectively utilizes gradient context assistant judgments to achieve higher accuracy and smoother effects with similar running times. Second, inspired by the importance of the initial value in complex nonlinear problems, an IVBP algorithm for improving both the convergence speed and accuracy is presented, which provides disparities with different probabilities using GF instead of equal probabilities. These methods improve both convergence speed and accuracy and are an exploration of the combination of local methods and global methods. The methods are evaluated with the Middlebury and new 2014 Middlebury datasets. Experimental results demonstrate that the proposed method maintains superior performance and holds better edge-preserving smoothing effects compared with some excellent BP algorithms, especially near disparity discontinuities and in textureless regions. Our IVBP is one of the best BP algorithms at present.

Future work should aim to incorporate more contextual measures, such as differential geometry properties, into the data term of the BP algorithm.
